# Supplementary material for: Botulinum neurotoxin A modulates the axonal release of pathological tau in hippocampal neurons
Source: Toxicon. 2023 Jun 1;228:107110. doi: 10.1016/j.toxicon.2023.107110 (PMC10636589; doi:10.1016/j.toxicon.2023.107110)
Supplement: Multimedia component 1 [file mmc1.pdf]

Supplementary material

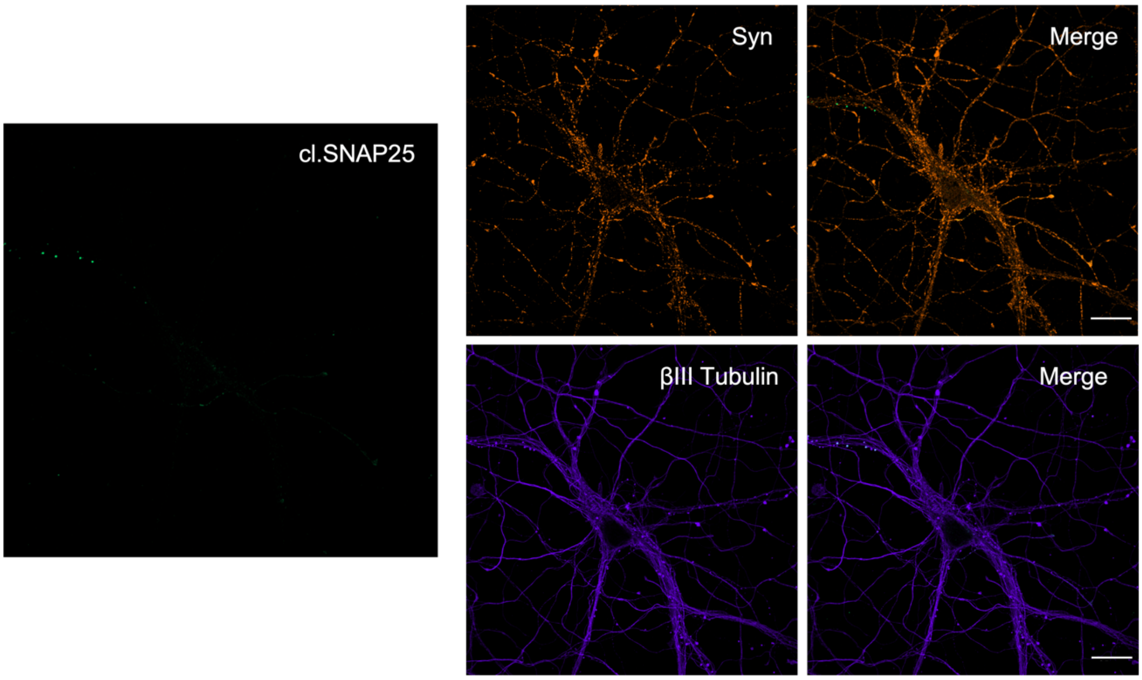

Figure 1. The anti-cl.SNAP25 antibody gives very low or no signal in non-treated neurons.

Representative images of DIV15 primary hippocampal neurons showing baseline signal of cleaved SNAP25 (cl.SNAP25, green) in the absence of BoNT/A. This signal was measured in synaptophysin (Syn, orange) and βIII tubulin (purple) positive areas of non-treated neurons to determine the specificity of the antibody. No significant signal was detected. Scale bar = 25 µm.

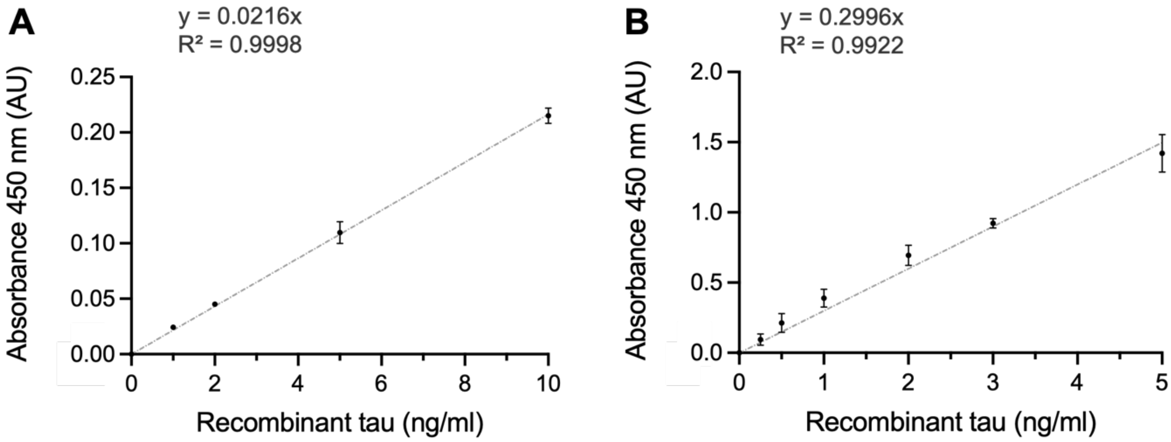

Figure 2. Sandwich ELISA optimisation for hTau detection in neuronal media.

Increasing concentrations of recombinant hTau were detected using a newly developed sandwich ELISA. Graphs show the absorbance at 450 nm as a function of increasing concentrations of hTau; the intercept was set to 0. A) Absorbance at 450 nm of recombinant hTau was detected using a secondary antibody conjugated with HRP. The signal detected for the lowest concentration used (1 ng/ml) of protein is just above zero, showing the low sensitivity of this method of detection. B) Absorbance at 450 nm of recombinant hTau was detected using a biotinylated secondary antibody and then streptavidin conjugated with poly-HRP. This led to an increase in sensitivity of the assay by about tenfold, compared to A. As a result, the lowest hTau concentration detected was 0.25 ng/ml.

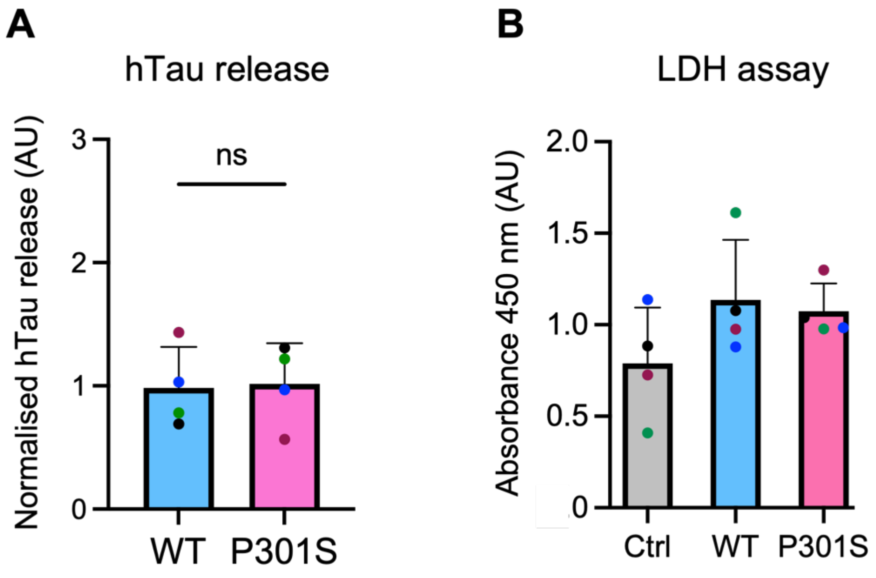

Figure 3. Equivalent amounts of WT and P301S mutant hTau are released in the culture media of unstimulated hippocampal neurons.

A) Similar levels of hTau are present in the culture media of resting DIV15 primary hippocampal neurons after 7 days of lentiviral expression of WT or P301S hTau. Unpaired Student's t-test was used to compare WT and P301S, and no significant difference was detected. n = 4. B) LDH assay of the somatic cell media of DIV15 neurons. The bar chart shows absorbance levels at 450 nm. A one-way ANOVA with Tukey's multiple comparisons test was performed, and no significant difference was detected. n = 4.
